# Supplementary material for: Creating a foundation for origin of life outreach: How scientists relate to their field, the public, and religion
Source: PLoS One. 2023 Feb 24;18(2):e0282243. doi: 10.1371/journal.pone.0282243 (PMC9956591; doi:10.1371/journal.pone.0282243)
Supplement: S1 Fig — How different communication profiles prioritize aspects of communication. (PDF) [file pone.0282243.s001.pdf]

Figure S1: Communication aspects priority for communication profiles

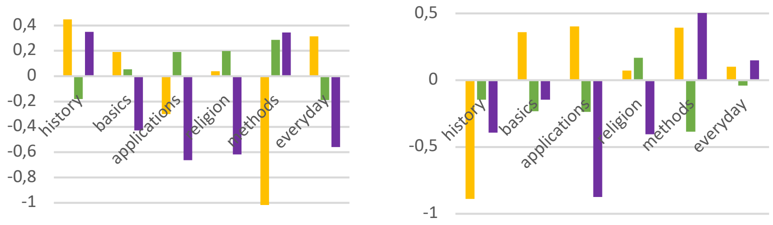

To compute the values in figure, we considered the average priority A given by researchers fitting the different profiles when communicating about the OoL controversy (left panel) or the one with religion (right panel). For each of these, we then compute the z-score:

$$z = (A - m) / S \,, \qquad (1)$$

Where m is the average priority score of that aspect and S the associated standard deviation (as reported in Table 1 in the main text. Researchers fitting the *teaching* profile for the OoL debate (yellow bars in the left panel, n=10) give higher priority to historical theories and everyday life of researchers than the average (N=46). Meanwhile, they would rather leave discussions about methods and processes of research. Scientists fitting the advocating profile (green bars, n=21) mostly conform to the average. However, they are the ones giving the highest priority to the relationship with religion, the only ones to give more than average priority to potential applications, and the only ones giving less than average priority to historical theories. Very few scientists fit the *discussing* profile (purple bars, n=3), which explains the wide deviations. They are the only ones giving less than average scores to scientific basics or relationship with religion.

The right panel presents analogous data, but using the communication profiles related to the controversy with religion. In this case, the *teaching* profile (yellow bars, n=5) shifts to lower than average priority for history, while switching to higher than average priority with regards to scientific basics and potential applications. Scientists fitting the *advocating* profile (green bars, n=10) give less than average priority to every aspect, except for the relationship with religion. The *discussing* profile (purple bars, n=3) is again the least populated. Scientists fitting this profile in the religion controversy have similar priorities as their counterparts in the OoL debate, except for a reduced interest in history and an increased priority given to the everyday work of researchers.
