# Supplementary material for: Creating a foundation for origin of life outreach: How scientists relate to their field, the public, and religion
Source: PLoS One. 2023 Feb 24;18(2):e0282243. doi: 10.1371/journal.pone.0282243 (PMC9956591; doi:10.1371/journal.pone.0282243)
Supplement: S2 Fig — Controversy and communication profiles related to the scientific controversy and the controversy with religion, segmented by field of work, hypothesis preference, seniority, religiosity, number of countries researchers lived in. (PDF) [file pone.0282243.s002.pdf]

Figure S2: Additional demographics for controversy and communication profiles

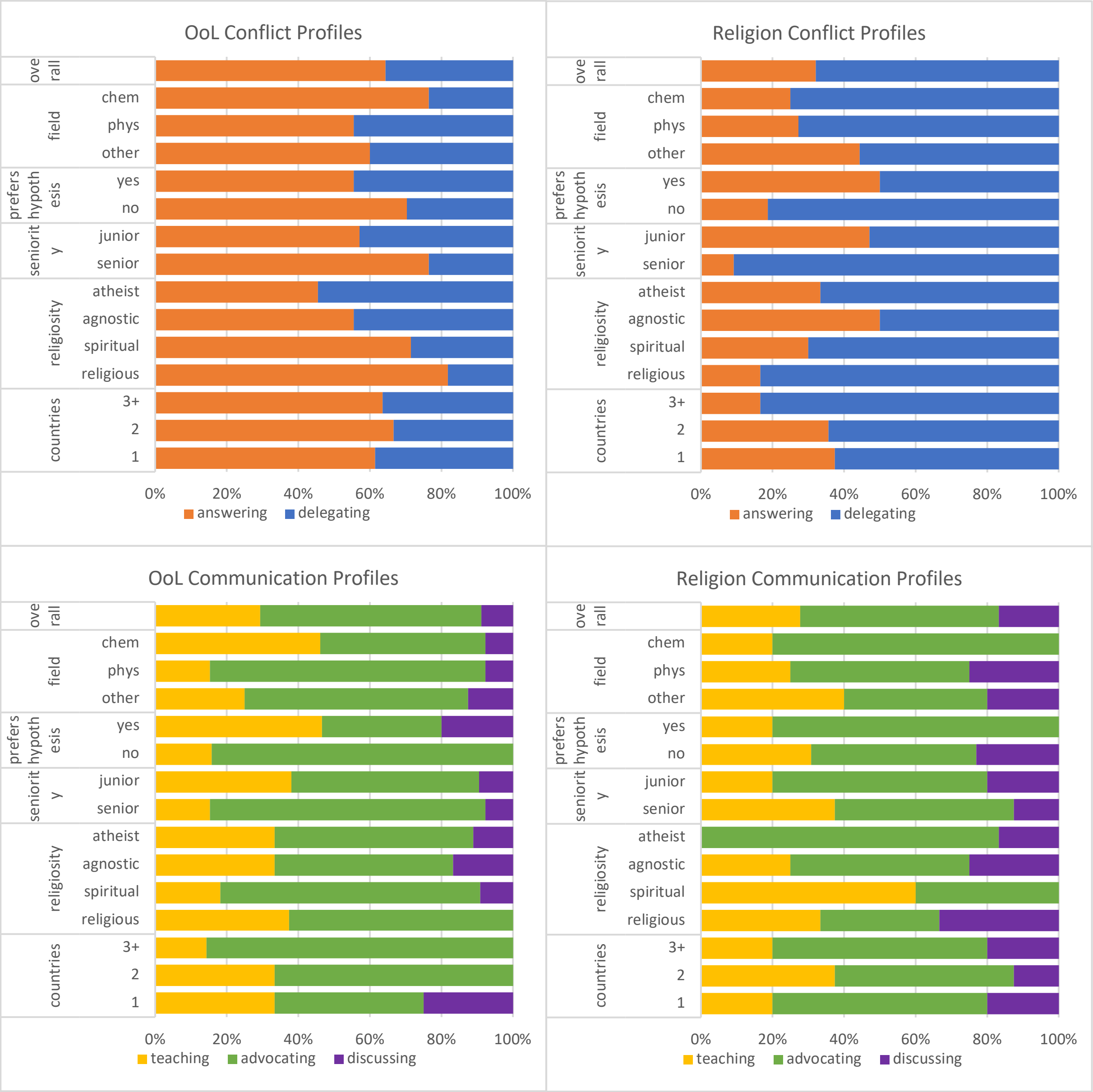

The top two panels show the distribution of the controversy profile for the OoL debate (left, n=45) and the for the controversy with religion (right, n=28) according to discipline, to whether the scientists expressed preference for any OoL-hypothesis, their career stage, religiosity and number of countries visited during their career.

The bottom two panels show analogous distribution of the communication profile on the OoL debate (left, n=34) and the debate with religion (right, n=18).
