# Supplementary material for: Creating a foundation for origin of life outreach: How scientists relate to their field, the public, and religion
Source: PLoS One. 2023 Feb 24;18(2):e0282243. doi: 10.1371/journal.pone.0282243 (PMC9956591; doi:10.1371/journal.pone.0282243)
Supplement: S1 Table — Classification table with rules that defined each of the classes and profiles. (PDF) [file pone.0282243.s003.pdf]

S1 Table: Rules to define classes

Cluster	Class	Definition	Rules	Quote
Actors of Controv. A	Groups 1	Groups or institutions collectively engage in the conflict.	One of:<br>• Institutions are in conflict with each other<br>• Groups are in conflict within or across institutions	And there's definitely teams, like Team A/Team B. But in general, […] I would say it's not so much a conflict of who believes in what as a conflict of who is what. [23]
	Individuals 2	Conflictual aspects only arise between individuals.	All of:<br>• Conflict limited by action of single individuals<br>• Small groups do not become institutional<br>• Individuals create or entrench conflict	It's always a history of person. And if two persons don't like them, they will go in conflict. If they respect themselves, it will be constructive, even though they have different opinions. [27]
Own Role in Controv. B	Judge 2	The interviewee wants to participate in determining which side of the debate is right.	All of:<br>• Provides or exchanges information<br>• Initially impartial<br>• Actively participates in conflict resolution	This is for me something where I can, hopefully very objectively, decide if a process is possible or not [35]
	Defender 1	The interviewee actively defends or advances a specific side in the conflict.	All of:<br>• Actively participates, taking a side<br>• Defends her side from attacks	If we do our jobs well we will be able to prove one day that the origin of life didn't have to be mystical, it didn't have to be created by God [9]
	Information Supplier 3	The interviewee only wants to provide information.	All of:<br>• Provides or exchanges information<br>• Impartial<br>• Delegates conflict resolution	I'm contributing valuable information to the topic. And whoever finally finds out which theory is the right one and gets the right answers, I hope that I contributed enough information for the final answer. [28]
	Bridge Builder 4	The interviewee wants to actively reduce the most conflictual aspects.	All of:<br>• Actively participates as peacemaker<br>• Convinces people to sit around the table<br>Defends her side from attacks	The whole point of this project is to gather amount of information from every field and then try to dig up and say, "Okay, so that's how it happened. Maybe." So I think you wouldn't be too conflictable [2]<br><br>if somebody wants to fit your science into his views, you maybe should help him. [4]<br><br>Why not to make connection with what was traditionally explained? Or what was the interpretation? That was given in that field? And what is the scientific explanation or interpretation, but also science is an interpretation of reality. [40]
Cause of Controv. C	Violation 2	Cause of conflict is active (possibly willing) violation of rules or boundaries by some participants.	• Conflict unnecessary<br>And one of:<br>• Actors willingly violate boundaries between institutions<br>• Institutions refuse to accept boundaries<br>• Scientists violate Mertonian rules	You need a couple of integrative figures […] You shouldn't be dogmatic about your research. And I think that's a lesson we have to learn. [8]<br>There's a lot of conflict, but there doesn't have to be. […] If there were to be no conflict, it would be religion who would have to accommodate. [9]
	Hard-wired 3	The conflict is a natural part of the process or of the actors involved.	One of:<br>• Conflict hard-wired in sense-making processes<br>• Inevitable conflict between different (but equally valid) value systems<br>• Correct answer is impossible to find	That's how we go from one point to more knowledge, right? To challenge the views, to challenge the beliefs, to challenge the established science. [21]
	None 4	There is no conflict—at most constructive dialogue.	One of:<br>• Different positions are complementary<br>• Parties engage in constructive dialogue	For me, science and the beauty of mathematics, of physics, theoretical physics, of how the world works, and how beautiful and how logical everything is, this is for me the best proof that there's some God. [5]
	Lack of knowledge 1	Some conflict actors are misinformed, lack understanding, or are unaware of boundaries they violate, causing the conflict.	• Conflict unnecessary<br>And one of:<br>• Some party has wrong information<br>• One or more value system wrong<br>• Some actors unable to come to right conclusions	If you think that God created the earth in seven days and all that crap, that's not gonna work. But if, on the other hand, you think that maybe God is more a force of nature or something, then yes. [11]

Cluster	Class	Definition	Rules	Quote
Public attitude D	Positive 1	The public is interested in the topic, has a favorable view of this field research.	One of:<br>• Curiosity or interest before communication<br>• Communication creates excitement<br>• Topic is relatable	They're super interested. They really want to know. [20]<br><br>Once you start telling 'em about what you're doing, then it becomes fascinating. [15]<br><br>Everyone, at least once wondered, "Where do I come from?" [1]
	Negative 3	The public has negative view of this field of research.	One of:<br>• Hostility to research<br>• Topic boring or too complex<br>• Public sides with opposing faction	I know that a lot of people are opposed to it [10]
	Unaware 4	This topic or this controversy is unknown to the public.	One of:<br>• Public does not know about research<br>• Little communication existing about topic	Over 95% of the population probably has no idea it exists [9]
	Indifferent 2	The public has neither positive nor negative views of research on this topic.	• Interest or attitude neither positive nor negative	I think they would, in the first place, do not understand why we do it. [28]
Target E	Generic 2	Communication is directed at a broad, undifferentiated public.	• No specific segment or target	Everyone, everyone, really? So from kids to grandmas? [4]
	Single Segment 1	Communication targets a specific segment of the public.	All of:<br>• One or few specific segments as target<br>• Uniform communication	I would have in mind people who go to a museum and are interested in a little bit of science. I wouldn't necessarily talk to people on the streets about my topic. [28]
	Diverse Segments 3	Communication deliberately addresses different segments, with dedicated messages or methods.	All of:<br>• Multiple specific target segments<br>• Communication differentiated by segment	Everybody that goes to a talk, to a presentation that is called "Origin of Life". And, yeah, also particular children. [5]
Prior knowl. F	Lacking 2	The public has less information than it should about this topic.	One of:<br>• Scarce information<br>• Public needs to know more	I think that many don't know, or are lacking basics of biology […]I think many people, when they hear proteins, they think about going to the gym [15]
	Enough 1	The public has "enough" information, can get information if wanted, does not need more.	One of:<br>• Information available if wanted<br>Public has information they need	If they're not curious to know, I think it probably is enough for them. [23]<br><br>I also don't really know what it would add to their lives if they knew more. [6]
	Wrong 3	The public is misinformed about the topic.	One of:<br>• Wrong information<br>• Amount of information anticorrelated to correctness	I've had my mother tell me, "Well, what is there to research? Like, you know, we know that! Like, we all like evolved from like amoebas." [9]<br><br>Certain part of community that likes to write books for the general public. And then people read those books, and they believe this is the consensus in the field.[8]

Model<br>G	Deficit<br>1	Communication model aimed at filling a knowledge gap between scientists and public.	<u>All of:</u><br>• One-directional communication<br>• Goal: filling knowledge gaps<br>• Decides what information is important	If we just make the everyday religious person understand that their God doesn't have to go away just because he didn't create life directly […], we might increase scientific literacy. And that's a good thing. [9]
	Dialogue<br>2	Communication involves a two-way flow of information, with public expressing views or requests.	<u>All of:</u><br>• Two-way communication (at least partially)<br>• Goal: satisfying curiosity<br>• Asks or listens to what information is important for audience	Of course, they will care about it. Like they will ask you, "How did you sample it? How did you take your samples?" [6]<br><br>They seem interested to know more. But I don't know where to start [11]<br><br>So you probably want to be smart about giving some information about the field, but still kind of interesting for the person listening to it, so "Okay, and then I want to know more."[13]
	Participation<br>3	Scientists and public cooperate to determine what is important to discuss.	<u>All of:</u><br>• Two-way communication<br>• Priorities and contents co-determined	Include the public in this discussion a bit. And yeah, let them have their own opinion on that. And also the question to which extent is relevant for our lives. [5]<br><br>I would totally argue with, you know, showing that there is dialogue and there's also, you know, disagreement, and then let them take sides. And let's see what, you know, what comes from stakeholders on the street, about, you know, what could it be important aspects. [19]
Role in Comm<br>H	Popularizer<br>4	The interviewee communicates only more understandable or "interesting" aspects.	<u>One of:</u><br>• Wants to satisfy public's curiosity<br>Controversy beyond public comprehension	It's a nice story you can tell. And it's a stepwise rise in complexity, because, like most cases, the science starts with a very simple idea, and more and more complex, so it's easy to introduce the basic and to build that up. [1]
	Advocate<br>1	The interviewee communicates to promote an institution or to defend a side in the controversy.	<u>One of:</u><br>• Wants to defend research in controversy<br>• Wants to promote image of research<br>Wants to promote institution of research	It's kind of embarrassing to show to the public how researchers—which are thought to be the authority on knowledge—are fighting over if this molecule is prebiotic or not. [30]<br><br>It's not dangerous, we're not trying to clone or something. [15]
	Reporter<br>3	The interviewee wants to presents facts as completely, impartially, and transparently as possible.	<u>One of:</u><br>• Wants or feels the duty to report to the public<br>• Presents topic as fully and transparently as possible	Just to have everyone on the same page, a nice summary of what is going on, from both sides, would be nice. [10]<br><br>I would probably pick sort of the three, four, sort of most discussed hypotheses, and then present them honestly, with the pros and cons. [8]<br><br>We should always make clear what are the scientific grounds? And where are we leaving the facts? Well, what are the conclusions? What are the possible conclusions? And what conclusions are not possible and just speculative? [19]<br><br>From a historical perspective, it's important to also... to consider more of these points. But you know—as a chemist, again—I would not consider these points as being relevant for my own experience. But in principle, historically spoken, it's... it's... of course it's important and one should consider and keep in mind [32]
	Peacemaker<br>2	The interviewee communicates to define or patrol boundaries between different groups or institutions.	• Wants to prevent or end conflict<br><u>And one of:</u><br>• Denounces encroachment of boundaries<br>• Dispels incorrectly perceived encroachments	I think already showing that the origin of life research is not just research to disprove that God exists [12]<br><br>To present this field to the public, I think it's important to stay within sciences that do use the scientific method and do not take just assumptions [22]
