# Supplementary material for: Creating a foundation for origin of life outreach: How scientists relate to their field, the public, and religion
Source: PLoS One. 2023 Feb 24;18(2):e0282243. doi: 10.1371/journal.pone.0282243 (PMC9956591; doi:10.1371/journal.pone.0282243)
Supplement: S2 Table — Composition of each controversy profile in terms of most frequent answer classes. (PDF) [file pone.0282243.s004.pdf]

S2 Table: Controversy profile definitions

Profile	Defining classes
**Delegating**	**Conflict Role** (cluster B) is:<br>“Information Supplier” (B3)<br>or<br>“Bridge Builder” (B4)<br>**OR**<br>**Cause of conflict** (cluster C) is<br>“None” (C4)
**Answering**	**Conflict Role** (cluster B) is:<br>“Defender” (B1)<br>or<br>“Judge” (B4)<br>**OR**<br>**Cause of conflict** (cluster C) is<br>“Hard-Wired” (C3)

After the answers were categorized, we looked in each interview for the most frequent class for each cluster (e.g. “which type of cause for conflict did the person cite most frequently?”).
The interviewee was then assigned a profile based on whether their most frequent answers fit any of these combinations. For example, if a person cited most frequently their role in the controversy as “information suppliers” or “bridge builders” or found no conflict happening, they would be assigned to the delegating profile.
