# Supplementary material for: Creating a foundation for origin of life outreach: How scientists relate to their field, the public, and religion
Source: PLoS One. 2023 Feb 24;18(2):e0282243. doi: 10.1371/journal.pone.0282243 (PMC9956591; doi:10.1371/journal.pone.0282243)
Supplement: S3 Table — Composition of each communication profile in terms of most frequent answer classes. (PDF) [file pone.0282243.s005.pdf]

S3 Table: Communication profile definitions

Profile	Defining classes
**Advocating**	**Communication role** (cluster H) is:<br>"Advocate" (H1)
**Teaching**	**Communication role** (cluster H) is:<br>"Popularizer" (H4)<br>**AND**<br>**Communication model** (cluster G) is<br>"Deficit" (G1)
**Discussing**	**Communication role** (cluster H) is:<br>"Reporter" (H3)<br>**AND**<br>**Communication model** (cluster G) is NOT<br>"Deficit" (G1)
