# Supplementary material for: Creating a foundation for origin of life outreach: How scientists relate to their field, the public, and religion
Source: PLoS One. 2023 Feb 24;18(2):e0282243. doi: 10.1371/journal.pone.0282243 (PMC9956591; doi:10.1371/journal.pone.0282243)
Supplement: Attachment — Submitted filename: Response_to_reviewers.docx [file pone.0282243.s007.docx]

München, 24.10.2022

Dear Editor,

Thank you very much once again for considering my paper for publication in PLOS ONE.

Based on the Editor and Reviewer’s suggestions and comments, we were able to extensively edit the Manuscript. We clarified, improved, and extended the discussions as much as possible. Moreover we substantially expanded the supplementary materials to address the concerns of more specialized and/or interested readers.

Below our answers to each of the Reviewer’s comments.

Sincerely,

Karl Wienand

- In line 47 the authors state that the majority of research on creationism has taken place in the US but they could also easily reference recent contributions on Europe.

  We thank the Reviewer for pointing this out. Indeed, limiting the discussion of creationism debates to the US environment could be reductive. We expanded the references (particularly Refs 14—16) to include the European context, as well as debates around non-Christian creationism.
- In lines 49-56 statistics are compared regarding scientists in Western Europe and the general German population- it would be better if the comparison groups were the same' ie German scientists w the German general population or Western European scientists with the general Western European population.

  We thank the reviewer for the comment. Comparing more uniform data provides a better picture of secularization among scientists. We researched different sources (i.e., Refs 20, 21) to compare our sample with Western European and EU general population.
- In lines 91-94 the religious identity of the scientists was described and it was not clear regarding those scientists of non-Christian backgrounds- there were no Muslim, Hindu or Jewish scientists? This needs some further elaboration.

  No, the sample happened to not include any scientists with Hindu or Jewish background. One was raised Muslim but no longer identifies as affiliated. We amended the statement for clarity, but left the part still intentionally vague to protect the participant’s anonymity.
- The material in lines 104-116 could be switched to a note

  These considerations, though admittedly not necessary to replicate the study, had to be included in the Methods section following a request from PLOS ONE.
- The sentences in lines 310-313 could be moved to an earlier section.

  We thank the reviewer for the suggestion. We added a similar text to the beginning of the Results section. However, we also left the original lines in place, as a quick recap for readers who may have skipped directly to the Discussion section.
- Some quotations brought from the interviews were not always clear

  We thank the reviewer for the suggestion. In preparing the manuscript we had tried to leave the quotes as much as possible in their original form. As the reviewer points out, however, this caused problems, as it was sometimes unclear what the interviewees were referring to with “it”, “they”, etc. We edited quotes throughout the manuscript for clarity. All alterations to the original transcripts are denoted in square brackets. For example, the cited quote in Table 3 now reads: “I think they would, in the first place, do not understand why we do [this research].”
- Some occasional lines in the text that should be adjusted (e.g. line 369)

  We thank the reviewer for the comment. We polished the entire text, of course starting from that line.
- More discussion of the categories would be helpful to explain the choices made by the researchers and to describe further the contours of the typology they found (eg the examples brought for single actors vs groups were more focused on the constructive nature of differing views where as those who saw camps seemed to describe this in competitive terms-- is this significant, coincidental etc?)

  We thank the reviewer for the keen interest in the topic and the thoughtful remark. The example quote for “Single actors” was chosen because it stresses how the conflictual nature depends, according to this interviewee, only on the people involved (“it's always a history of person”). We expanded the manuscript text to clarify this example as well as the overall category.
  We also agree that interested readers should have access to a more detailed treatment of our classification process and rules. During the analysis we worked with a more comprehensive table than the one shown in the manuscript. This complete classification table included specific a priori rules that determined to which class a sentence would be assigned. While we still believe the summarized table in the main manuscript is more accessible for the broad readership of PLOS ONE, we agree that it may leave the boundaries too blurry for the interested reader. Therefore we added to the supplementary material the full rule table (S1 Table) that was used to analyze the interview content, as well as the rules delineating each the controversy and communication profiles (Tables S2 and S3, respectively).
